# The Role of Unimodal Feedback Pathways in Gender Perception During Activation of Voice and Face Areas

**DOI:** 10.3389/fnsys.2021.669256

**Published:** 2021-05-28

**Authors:** Clement Abbatecola, Peggy Gerardin, Kim Beneyton, Henry Kennedy, Kenneth Knoblauch

**Affiliations:** ^1^Univ Lyon, Université Claude Bernard Lyon 1, INSERM, Stem Cell and Brain Research Institute U1208, Bron, France; ^2^Centre for Cognitive Neuroimaging, Institute of Neuroscience and Psychology, University of Glasgow, Glasgow, United Kingdom; ^3^Institute of Neuroscience, State Key Laboratory of Neuroscience, Chinese Academy of Sciences Key Laboratory of Primate Neurobiology, Shanghai, China; ^4^National Centre for Optics, Vision and Eye Care, Faculty of Health and Social Sciences, University of South-Eastern Norway, Kongsberg, Norway

**Keywords:** psychophysics, conjoint measurement, functional magnetic resonance imaging, dynamic causal modeling, gender comparison, predictive coding

## Abstract

Cross-modal effects provide a model framework for investigating hierarchical inter-areal processing, particularly, under conditions where unimodal cortical areas receive contextual feedback from other modalities. Here, using complementary behavioral and brain imaging techniques, we investigated the functional networks participating in face and voice processing during gender perception, a high-level feature of voice and face perception. Within the framework of a signal detection decision model, Maximum likelihood conjoint measurement (MLCM) was used to estimate the contributions of the face and voice to gender comparisons between pairs of audio-visual stimuli in which the face and voice were independently modulated. Top–down contributions were varied by instructing participants to make judgments based on the gender of either the face, the voice or both modalities (*N* = 12 for each task). Estimated face and voice contributions to the judgments of the stimulus pairs were not independent; both contributed to all tasks, but their respective weights varied over a 40-fold range due to top–down influences. Models that best described the modal contributions required the inclusion of two different top–down interactions: (i) an interaction that depended on gender congruence across modalities (i.e., difference between face and voice modalities for each stimulus); (ii) an interaction that depended on the within modalities’ gender magnitude. The significance of these interactions was task dependent. Specifically, gender congruence interaction was significant for the face and voice tasks while the gender magnitude interaction was significant for the face and stimulus tasks. Subsequently, we used the same stimuli and related tasks in a functional magnetic resonance imaging (fMRI) paradigm (*N* = 12) to explore the neural correlates of these perceptual processes, analyzed with Dynamic Causal Modeling (DCM) and Bayesian Model Selection. Results revealed changes in effective connectivity between the unimodal Fusiform Face Area (FFA) and Temporal Voice Area (TVA) in a fashion that paralleled the face and voice behavioral interactions observed in the psychophysical data. These findings explore the role in perception of multiple unimodal parallel feedback pathways.

## Introduction

Hierarchical processing has long played a prominent role in understanding cortical organization and function (Hubel and Wiesel, 1962, 1965; Rockland and Pandya, 1979; Felleman and Van Essen, 1991; Ferster et al., 1996; Angelucci et al., 2002; Angelucci and Bressloff, 2006). Feedforward models of receptive field construction have been recently complemented by the integration of structure and function in large-scale laminar models of feedback/feedforward processes engaged in cortical hierarchy (Kravitz et al., 2013; Baldauf and Desimone, 2014; Markov et al., 2014). The relevance of connectivity based structural hierarchy (Markov et al., 2014) is reinforced by its remarkable agreement with functional hierarchy derived from inter-areal causal relations based on synchronization of different frequency bands of the local field potential obtained with surface electrodes in macaque (Bastos et al., 2015) and magnetoencephalography recordings in human (Michalareas et al., 2016). Feedback processes have been studied in relation to top–down generative networks involved in attention, perceptual integration, prediction, perceptual learning, mental imagery and consciousness (Roelfsema and de Lange, 2016; Van Vugt et al., 2018). Our understanding is further complexified by the fact that functional connectivity cannot be described uniquely in terms of a single pair of feedback/feedforward channels (Gerardin et al., 2018; Bergmann et al., 2019). A multiplicity of functional pathways is supported anatomically, an example of such being the dual counterstream architecture (Markov and Kennedy, 2013; Barzegaran and Plomp, 2021; Federer et al., 2021; Vezoli et al., 2021).

Despite spectacular conceptual progress in the context of predictive coding that conceives of feedforward pathways as transmitting prediction errors and feedback pathways predictions (Clark, 2013), Jean Bullier’s question “What is fed back” (Bullier, 2006) remains as urgent today as it was when he formulated it. This is largely because experimentally it is considerably more difficult to invasively manipulate the top–down processes that come under the banner of predictions than it is to record the consequences of changes in the sensorium on bottom-up processes (Zagha, 2020; Vezoli et al., 2021). In this context, sensory integration provides an ideal model system to investigate feedback processes. Here, we have examined face and voice interactions in a gender comparison task.

Because face and voice perception allow the retrieval of similar information about others (e.g., identity, gender, age, emotional state, etc.) and engage similar brain mechanisms, it has been proposed that they share a privileged link (Campanella and Belin, 2007; Yovel and Belin, 2013; Hasan et al., 2016; Belin, 2017). In particular the Fusiform Face Area (FFA), a functionally defined region in the temporal fusiform gyrus, has been shown to respond strongly to faces (Kanwisher et al., 1997; Kanwisher and Yovel, 2006), while the Temporal Voice Area (TVA), a region in the lateral temporal cortex, has been proposed to be the equivalent of the FFA for voices (Belin et al., 2000; von Kriegstein and Giraud, 2004; Pernet et al., 2015).

Face-voice gender recognition is robust and precocious, appearing as early as 6–8 months in human development (Walker-Andrews et al., 1991; Patterson and Werker, 2002). The TVA and FFA are both involved in the unimodal recognition of gender in their respective modalities (Contreras et al., 2013; Weston et al., 2015). In addition, both of these areas along with a supramodal fronto-parietal network have been found to be activated during face-voice gender categorization with a functional magnetic resonance imaging (fMRI) protocol (Joassin et al., 2011). Importantly from a psychophysical perspective, and contrary to other face-voice properties such as emotion, gender is defined along a single perceptual dimension (varying between masculine and feminine), simplifying analyses of responses. Technically, this makes it possible to generate a continuous physical variation between male and female stimuli via morphing of auditory and visual stimuli (Macke and Wichmann, 2010; Watson et al., 2013).

One study found that incongruent face cues reduced the proportion of correct gender categorizations of voices, but incongruent voice cues did not influence gender categorization of faces (Latinus et al., 2010). In contrast, Watson et al. (2013) observed using audio and video morphing that voices were more disruptive for face judgments than were faces for voices, which they attributed to the greater dimorphism with respect to gender of voices compared to faces. Both studies used a single-stimulus gender identification paradigm to measure the probability of a male/female decision. To access face and voice contributions on a common scale using only probabilities it is necessary to model the common underlying decision process. Additionally, while Watson et al. (2013) were able to detect cross-modal effects, they were unable to describe the rules by which voice and face cues are combined qualitatively and quantitatively. We can obviate these problems by using the psychophysical method of maximum likelihood conjoint measurement (MLCM) that links gender comparisons to a signal detection model of the decision process. This approach formalizes a set of testable nested models describing several types of face-voice combination rules (Ho et al., 2008; Knoblauch and Maloney, 2012; Maloney and Knoblauch, 2020).

In the present report, we examine how visual and auditory signals are exploited with respect to gender perception, a high level feature of voice and face perception. In both the visual and auditory systems, auditory and visual signals, respectively, provide a readily identifiable top–down mechanism for cross-modal effects (Petro et al., 2017). Here, we use a psychophysical task to evaluate models of top–down and bottom–up signaling in multi-modal interactions. In order to clarify the neural correlates of the perceptual processes, we employ functional imaging to elucidate the effective connectivity underlying the behavioral responses.

## Materials and Methods

### Psychophysics

#### Design and Procedure

**FIGURE 1:**
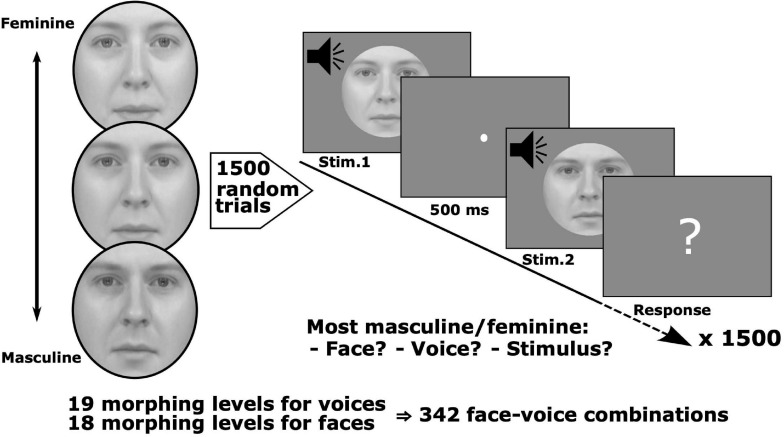
Stimulus set and conjoint measurement protocol. Pairs of face-voice video sequences with independently varying levels of face and voice gender morphing were judged by observers according to: (1) face gender, (2) voice gender, or (3) stimulus gender, i.e., taking both face and voice into account. Stimulus pairs were sampled from a set with 18 levels of morphing for the face and 19 levels for the voice. Six groups of observers judged which face, voice or stimulus was either more masculine or feminine (6 observers/group, each group balanced with respect to gender, 36 observers total, and 1,500 trials/observer).

Maximum likelihood conjoint measurement (Ho et al., 2008; Knoblauch and Maloney, 2012) was used to estimate the voice and face contributions to gender comparisons between stimulus pairs that covaried independently in the gender of the face and voice. Stimuli that varied gradually in gender along both modalities were constructed by morphing average male and female faces and voices, respectively (described in Watson et al., 2013; see section “Materials and Methods”). In separate sessions judgments were based either on the face, the voice or both components (Figure 1). Intuitively, if over many trials the relative frequency of choosing one face (voice) as more masculine (feminine) depends on the face (voice) gender difference between the two stimuli presented, independently of the voice (face) gender difference, then we conclude that the information from the voice (face) does not influence the face (voice). If such independence is violated then we model the mutual influences of the modality specific gender signals.

Thirty-six observers (18 male) with normal or corrected-to-normal vision volunteered for the experiment (mean age ± SD: 25.9 ±3.6 years). Each was randomly assigned to one of six experimental conditions so that there were 3 male and 3 female observers per condition. All observers but one (author PG) were naive, all were right handed, native French speakers. All observers had normal (or corrected to normal) vision as assessed by the Freiburg Visual Acuity and Contrast Test (FrACT) (Bach, 2007), and the Farnsworth F2 plate observed under daylight fluorescent illumination (Naval Submarine Medical Research Laboratory, Groton, CT, United States). Normality of face perception was assessed by the Cambridge Face Memory Test (CFMT) (Duchaine and Nakayama, 2006). All observers gave informed consent.

Experiments were performed in a dark room where the only light source was the stimulus display to which observers were adapted. Stimuli were displayed on an Eizo FlexScan T562-T color monitor (42 cm) driven by a Power Mac G5 (3 gHz) with screen resolution 832 × 624 pixels and run at a field rate of 120 Hz, non-interlaced. Calibration of the screen was performed with a Minolta CS-100 Chromameter. Observers were placed at a distance of 57.3 cm from the screen. Head stabilization was obtained with a chin and forehead rest. Auditory stimuli were presented through headphones (Sennheiser HD 449), which also served to mask any ambient noise. Sound calibration was performed with a Quest QE4170 microphone and a SoundPro SE/DL sound level meter.

The stimulus set, obtained from Watson et al. (2013), consisted of video clips of a person saying the phoneme “had,” and whose face and voice varied by morphing from feminine to masculine (18 levels of morphing for the face and 19 levels for the voice yielding a total of 342 combinations). Clips were converted to greyscale and matched for luminance. An oval mask fitted around each face hid non-facial gender cues, such as the hair and the hairline (see Figure 1 for examples).

The software PsychoPy v1.80.07 (Peirce, 2008) controlled stimulus presentation. Stimuli were displayed in the center of a gray background (31.2 cd/m^2^, CIE *xy* = 0.306, 0.33). Face luminance varied between 29.7 cd/m^2^ (CIE *xy* = 0.306, 0.324) for the eyes and 51.6 cd/m^2^ (CIE *xy* = 0.303, 0.326) for the nose for all stimuli. Face diameter was fixed at 10 degrees of visual angle and voice volume between 85.2 and 86.7 dB SPL (A) – Peak.

Observers were tested over five sessions of 300 trials, yielding a total of 1,500 trials per observer [2.57% random subsampling from the ((19 × 18 – (19 × 18 – 1))/2 = 58,311 total number of non-matching, unordered pairs]. Given the large number of possible pairs tested, a subsampling paradigm was necessary to make tractable the number of trials for which each subject was tested. As this is a novel approach for performing MLCM experimentally, simulations justifying this procedure are presented in Supplementary Section 1. On each trial two stimuli were randomly selected, assigned and successively presented (Figure 1). The stimulus duration was fixed at 500 ms with a 500 ms inter-stimulus interval between each pair. After the presentation, observers were prompted to make a judgment comparing the genders of the two stimuli. The next pair was presented following the observer’s button press response. Observers were randomly assigned to one of six groups where each group was composed of equal numbers of males and females. Groups were defined by instructions to judge on the basis of the face, the voice or the stimulus, i.e., in the latter no specific instruction regarding modality, and whether they were instructed to choose which of the pair was more masculine or feminine. Six observers were tested in each of the 6 conditions.

#### Maximum Likelihood Conjoint Measurement

Maximum likelihood conjoint measurement aims to model the decision process of observers comparing multidimensional stimuli in order to determine how the observer integrates information across dimensions to render a judgment. Because the decision process is noisy, a signal detection framework is used (Ho et al., 2008) and the resulting model formalized as a binomial Generalized Linear Model (GLM) (Knoblauch and Maloney, 2012). Several nested models, corresponding to increasingly complex decision rules for combining information across modalities, are fitted to the data using maximum likelihood so as to maximize the correspondence between model predictions and observer decisions. These models are compared using nested likelihood ratio tests to determine the degree of complexity required to describe the observer’s decisions.

For example, consider two face-voice stimuli defined by their physical levels of morphing, [$\phi_{i}^{V}\,\phi_{i}^{A}$ for stimulus 1 or 2], for visual and auditory gender, S1 ($\phi_{1}^{V}\,\phi_{1}^{A}$) and S2 ($\phi_{2}^{V}\,\phi_{2}^{A}$), and the task of deciding whether the first or second stimulus has the more masculine face, i.e., the visual task. The noisy decision process is modeled as:

(1) $$\Delta \,\left(S_{1},S_{1}\right)=\psi_{1}-\psi_{2}+\epsilon =\psi \,\left(\phi_{1}^{V},\phi_{1}^{A}\right)-\psi \,\left(\phi_{2}^{V},\phi_{2}^{A}\right)+\epsilon =\delta +\epsilon$$

where *ψ*_1_ and *ψ*_2_ are internal representations for the gender of the first and second face, respectively, determined by the psychophysical function, ψ, ϵ is a Gaussian random variable with mean μ=0 and variance σ^2^ and Δ is the decision variable. We assume that the observer chooses the first stimulus when Δ > 0, and otherwise the second. The log-likelihood of the model over all trials given the observer’s responses is given by:

(2) $$\ell \,\left(\Delta_{i},R_{i}\right)\,\sum_{i}R_{i}\,\text{log}\,\left(\phi \,\left(\delta /\sigma \right)\right)+\left(1-R_{i}\right)\,\text{log}\,\left(1-\phi \,\left(\delta /\sigma \right)\right),$$

where *R_i_* is the response on the ith trial and takes the value of 0 or 1 depending if the subject chooses the first or second stimulus and Φ is the cumulative distribution function for a standard normal variable. For each model described below, the psychophysical responses, ψ′*s* were estimated that maximized the likelihood of the observer’s responses across all trials, with constraints imposed to render the model identifiable (Knoblauch and Maloney, 2012).

Under the independence model the observer exclusively relies on visual information and we define the decision variable:

(3) $$\Delta =\psi_{1}^{V}-\psi_{2}^{V}+\epsilon$$

where $\psi_{1}^{V}\,\psi_{2}^{V}$ are the internal representations of gender evoked by the visual cues of stimulus 1 and 2, respectively. A similar model is defined to model independent responses for the auditory task where the *V*’s are replaced by *A*’s.

In the additive model we define the decision variable as a sum of the visual and auditory gender signals.

(4) $$\Delta =\left(\psi_{1}^{V}-\psi_{2}^{V}\right)=\left(\psi_{1}^{A}-\psi_{2}^{A}\right)+\epsilon$$

where the visual and auditory terms of the equation have been regrouped to demonstrate that the observer is effectively comparing perceptual intervals along one dimension with perceptual intervals along the other (Knoblauch and Maloney, 2012).

Under the interaction model, non-additive combination terms are introduced. The decision variable can be written as

(5) $$\Delta =\left(\psi_{1}^{V}-\psi_{2}^{V}\right)+\left(\psi_{1}^{A}-\psi_{2}^{A}\right)+\left(\psi_{1}^{V\,A}-\psi_{2}^{V\,A}\right)+\epsilon$$

where the third term on the right side corresponds to an interaction that depends on each face-voice combination. This interaction model is usually non-specific as one term is evaluated independently for each combination of visual and auditory gender levels. Here, our results, described below, indicated that the additive terms could be characterized as parametric functions of the gender levels, *f*^*V*^(*ϕ*)and *f*^*A*^(*ϕ*), for visual and auditory modalities, respectively. This allowed us to test two specific types of interaction.

The *Congruence Interaction Model* introduces an internal congruence effect between face and voice gender within stimulus to yield the decision variable:

(6) $$\Delta =\left(f^{V}\,\left(\phi_{1}\right)-f^{V}\,\left(\phi_{2}\right)\right)+\left(f^{A}\,\left(\phi_{1}\right)-f^{A}\,\left(\phi_{2}\right)\right)+(|f^{V}\left(\phi_{1}\right)-f^{A}\left(\phi_{1}\right)|+|\left(f^{V}\left(\phi_{2}\right)-f^{A}\left(\phi_{2}\right)|\right)+\epsilon ,$$

This interaction depends on the absolute gender difference between visual and auditory signals for each stimulus. It has minimal effect on judgments when for each stimulus, the gender scale values are the same for both modalities and maximal when there is the greatest gender difference between modalities.

The *Magnitude Interaction Model* introduces a multiplicative effect of gender information across stimuli for the following decision variable:

(7) $$\Delta =\left(f^{V}\,\left(\phi_{1}\right)-f^{V}\,\left(\phi_{2}\right)\right)+\left(f^{A}\,\left(\phi_{1}\right)-f^{A}\,\left(\phi_{2}\right)\right)+\left(f^{V}\,\left(\phi_{1}\right)-f^{V}\,\left(\phi_{2}\right)\right)\cdot \left(f^{A}\,\left(\phi_{1}\right)-f^{A}\,\left(\phi_{2}\right)\right)+\epsilon .$$

This interaction is minimal when the gender difference between stimuli within either or both modalities is small and maximal when the within modality difference is large for both stimuli. Over many trials these differences cancel out more for stimuli that are closer to gender-neutral, so overall these gender-neutral stimuli will be associated with smaller effects for this interaction.

In other words, under the congruence interaction model, non-additive effects are assumed to be proportional to the *absolute* difference between face and voice gender. Under the magnitude interaction model, non-additive effects are assumed to be *proportional* to the amount of masculinity/femininity in the face and voice as compared to gender-neutral.

All psychophysical data were analyzed using R (R Core Team, 2019) and the package lme4 (Bates et al., 2015) to take inter-individual variability into account with generalized mixed-effects models (GLMM) (Pinheiro and Bates, 2000).

#### Signal Detection Theory and Optimal Cue Integration

The MLCM approach above describes the measuring and modeling of how attributes from different dimensions are combined in judgments of appearance. In this section, we explore relations to optimal cue combination, which is typically studied in the context of discrimination, and describe how these models lead to a more detailed consideration of the interaction terms in MLCM models.

The fusion of several cues into a single percept can be described using two classes of models (Clark and Yuille, 2013). In weak fusion models each cue is first processed separately in distinct and independent modules, before undergoing rule-based combination. By contrast, strong fusion models de-emphasize modularity and allow interaction effects at any processing level. The modified weak observer model (Young et al., 1993; Landy et al., 1995) that was established as a variant of weak fusion includes; (i) early interactions to make cues commensurable, whereby psychological variables, ψ_V_ and ψ_A_ along dimensions subscripted as *V* and *A* for visual and auditory, are expressed in the same units on the same internal perceptual axis directly allowing summation; (ii) the possibility of other potential interaction effects when the discrepancy between individual cues goes beyond that typically found in natural scenes, e.g., constraining physical values, *ϕ*_*V*_ and *ϕ*_*A*_, such that ψ_V_≈ψ_A_ to prevent such effects; (iii) a weighted average cue combination rule with weights dynamically chosen to minimize variance of the final estimate. This leads to the following signal detection model of the psychological response:

(8) $$\psi =N\,\left(\frac{\mu_{v}\cdot \sigma_{v}^{-2}+\mu_{A}\cdot \sigma_{A}^{-2}}{\sigma_{v}^{-2}+\sigma_{A}^{-2}},\frac{\sigma_{v}^{-2}\cdot \sigma_{A}^{-2}}{\sigma_{v}^{-2}+\sigma_{A}^{-2}}\right)$$

where ψ_S_ is the perceived level of the fused stimulus, distributed normally with μ_i_ the mean perceived level of the stimulus along dimension *i* (i.e., *V* or *A* depending on the modality), and $\sigma_{\text{i}}^{2}$ of the variance of the perceived level along dimension *i* (for derivation and a fuller description of this model see Supplementary Section 2).

This model leads to increases in variance causing the variance and the mean of the combined percept to be closer to whichever estimate is most reliable. The combined percept is expected to be more precise than either of the isolated unimodal estimates. Note that the observer is predicted to make binary decisions about combined stimuli, i.e., to classify them as either feminine or masculine in proportion to the position of the density distribution along the perceived gender axis with respect to a gender-neutral value.

Observers who use this maximization rule with respect to reliability are referred to as being statistically optimal and this type of response has been empirically verified in several domains for human multimodal integration (Ernst and Banks, 2002; Alais and Burr, 2004; Hartcher-O’Brien et al., 2014).

As already mentioned, one limitation of the maximization model is its dependency on the condition ψ_V_≈ψ_A_. In other words complementary interaction effects are expected to come into play in the case of inconsistencies beyond the discrimination threshold between the cues. In the absence of such interaction effects, the resulting percept is predicted to be the same when combining visual and auditory cues as long as the variances and $\frac{\psi_{\text{V}}+\psi_{\text{A}}}{2}$ remain constant.

An extension of the model includes interaction effects of congruence. When for example, one modality is very masculine and the other very feminine, we hypothesize that the stimulus is perceived as being less reliable than congruent stimuli. Consequently the variance of each unimodal estimate is increased in proportion to the distance between modalities in gender space before applying the optimal integration scheme. This has the effect of lowering the variance of the combined gender estimate, which biases the decisions because a higher proportion of the distribution falls on one side of the gender neutral reference.

Beyond this effect of congruence, it is conceivable that there is an effect of the magnitude of the gender difference from neutrality contained in each modality. For example, a face that is very clearly masculine might generate a more precise representation than a more gender-neutral face. Such effects would become more significant with greater incongruence between cues due to an increase in the difference in precision, and once again the resulting change in variance of the final estimate biases decisions.

The above models can be implemented by introducing an internal congruence parameter and/or a multiplicative effect of gender in order to account for the quality of gender information.

### Functional Magnetic Resonance Imaging

#### Design and Procedure

Twelve observers (6 female, mean ± SD age: 23.3 ± 3.3 years) participated in the study. All were right handed and screened for normal vision with the same battery of tests used in experiment 1. The study was approved by the local ethics committee (ID-RCB 2015-A01018-41). In accordance with the protocol, each subject was pre-screened by a clinician for suitability to undergo an fMRI procedure and gave informed consent. All participants attended two MRI sessions of approximately 1.5 h each and were remunerated for their participation.

All experiments were conducted using a MAGNETOM Prisma 3T Siemens MRI scanner at the Lyon MRI facility PRIMAGE, France. For each individual, high−resolution T1−weighted structural image (acquisition matrix 256 × 256 × 192, TR/TE: 3,500/3.42 ms, flip angle: 8°, resolution: 0.9 × 0.9 × 0.9 mm) and T2^∗^-weighted functional images (acquisition matrix 78 × 78, 49 slices, TR/TE: 2,500/26 ms, flip angle: 90°, resolution: 2.7 × 2.7 × 2.7 mm) were collected with a 64 channel head/neck Siemens coil. In order to control participants wakefulness, the left eye was monitored with an SR Research EyeLink 1000 Plus. Sound was provided using NordicNeuroLab earplugs. As sound stimulation was provided on top of scanner noise, we checked with each participant that they could hear the voices properly. This was also controlled quantitatively for some participants who were asked to perform a voice gender recognition task (detailed below). Subject responses were recorded using a Current Designs 904 diamond shaped 4 button device.

Stimuli were selected from a reduced set of those used in experiment 1 (Watson et al., 2013), consisting of video clips of a person saying the phoneme “had.” We used three levels of morphing for the face and voice (the most feminine, gender neutral and masculine in terms of physical morphing in both cases) generating a total of nine combinations. Software PsychoPy v1.84.2 (Peirce, 2008) was used to control stimulus presentation. Stimuli were displayed in the center of a gray background with a resolution of 1,024 × 768 pixels. Face diameter was fixed at 10° of visual angle.

Face-voice stimuli were presented in an event-related protocol. Each stimulus lasted 0.5 s and was followed by a fixation point with a random duration (5, 5.5, 6, 6.5, and 7 s). To prevent habituation we ensured that no more than three successive repetitions occurred of the same stimulus sequence (e.g., AAA), pair of stimuli (e.g., ABABAB), and fixation point intervals.

In order to mirror the tasks of the MCLM experiment, task-dependent instructions were given to attend to the gender of the face, the voice or the stimulus and no specific instruction regarding modality. Unlike the psychophysical paradigm, only a single face/voice combination was presented and therefore, no stimulus comparison was required. Attentional focus was controlled by randomly distributed response trials after which participants were prompted by a screen with a red feminine sign on one side and a blue masculine sign on the other (masculine and feminine sides varied randomly to prevent motor anticipation). They then had 2s to push a button on the left or the right side of a response device to indicate the gender perceived for the modality to which they had been instructed to attend.

**FIGURE 2:**
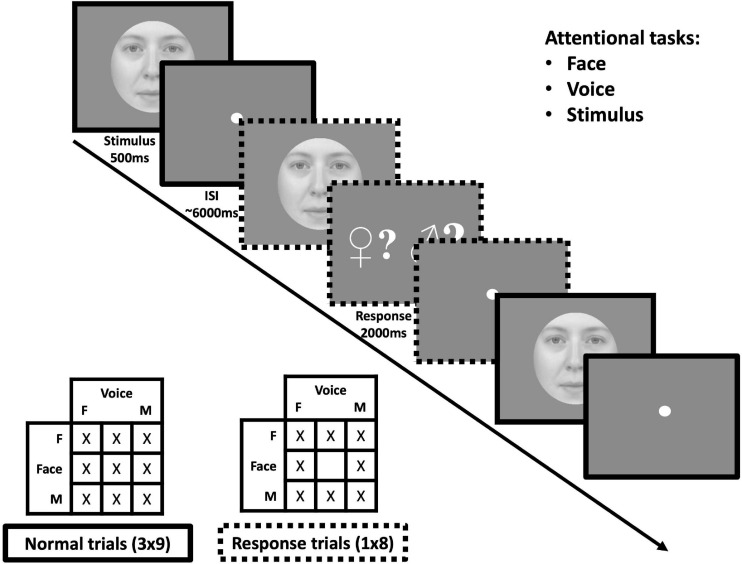
Protocol for one acquisition of the face-voice gender fMRI experiment. **(Top-right)** Within sessions, subjects were assigned the task to pay attention to face, voice or stimulus gender. **(Middle)** subjects were presented with face-voice stimuli in succession in an event-related manner. On some trials, subjects received a signal to respond as to whether the gender of the modality attended was masculine or feminine for the previous stimulus (note that in the actual protocol the masculine sign was presented in blue and the feminine sign in red). **(Bottom-left)** For each acquisition all nine face-voice gender combinations were presented three times in addition to eight “response” trials corresponding to all combinations except gender-neutral face + gender neutral voice.

During each acquisition, the nine gender combinations were presented three times in addition to eight response trials composed of every combination except a neutral face and a neutral voice (which would not be informative about the attentional state of the participant), see Figure 2. All participants performed two tasks (face and voice, face and stimulus or voice and stimulus) in two sessions separated by at least 1 day, each session being composed of five acquisitions. The total number of repetitions for each condition was 15 for all face-voice combinations and 40 response trials (for which the responses, but not brain activity, were analyzed to avoid motor contamination of fMRI data). Controlling for the order of the sessions (e.g., face followed by voice task or voice followed by face task) resulted in six possible pairs, each assigned to one male and one female participant. We thereby acquired 8 sessions in total for each task.

We identified two regions of interest (ROI) using functional localizers. During the first session we used a localizer for the FFA described by Pitcher et al. (2011) and Julian et al. (2012), in which subjects were presented with blocks of dynamic visual stimuli belonging to several categories: human faces, human body parts (hands, legs, etc.), objects, landscapes or scrambled images (created by spatially shuffling object videos subdivided by a 15 by 15 box grid). We reasoned that this dynamic protocol would reveal functional areas involved in our dynamic face-voice stimuli. In the second session, we used a localizer for the TVA described by Belin et al. (2000), in which subjects were presented with blocks of silence or auditory stimuli which were either vocal sounds (both with and without language) or non-vocal sounds.

Imaging data were first analyzed with Brain Voyager QX (Goebel, 2012). Preprocessing functional data consisted of slice-scan time correction, head movement correction, temporal high-pass filtering (two cycles) and linear trend removal. Individual functional images were aligned to corresponding anatomical image. These images were then used for 3D cortex reconstruction and inflation. No spatial smoothing was applied.

Fusiform Face Area and TVA were identified using a General Linear Model analysis including fixation periods and movement correction parameters as nuisance covariates. FFA was defined, bilaterally, as the set of contiguous voxels in the temporal fusiform gyrus that showed the highest activation for faces compared to body parts, landscapes, objects and scrambled images (Pitcher et al., 2011; Julian et al., 2012). TVA was defined, bilaterally, as the set of contiguous voxels in the lateral temporal cortex that showed the highest activation for vocal compared to non-vocal sounds (Belin et al., 2000). In both cases we used a significance threshold of *p* < 0.05 following false discovery rate correction. There is no reason to assume these functional areas are the same size across subjects but we checked that there were no outliers in terms of number of voxels (defined as a localized area that deviated from the mean number of voxels by greater than two standard deviations for an individual subject compared to the others).

#### Dynamic Causal Modeling

To explore the neural substrate of the multi-modal integration revealed by the psychophysical experiments, we performed a series of functional imaging experiments in which we evaluated the effective connectivity between areas implicated in processing face and voice stimuli. In particular, we restricted consideration to two candidate areas, FFA (Kanwisher et al., 1997; Kanwisher and Yovel, 2006) and TVA (Belin et al., 2000; von Kriegstein and Giraud, 2004; Pernet et al., 2015). FFA and TVA are ostensibly unimodal modules involved in face and voice processing. We translated the MLCM models described above into hypothetical effective connectivity networks in order to test whether non-additive interactions involve unimodal areas or exclusively occur at higher levels. Importantly, observing changes in effective connectivity between the FFA and TVA is agnostic to the role of direct communication between these areas or mediation by other top–down influences.

**FIGURE 3:**
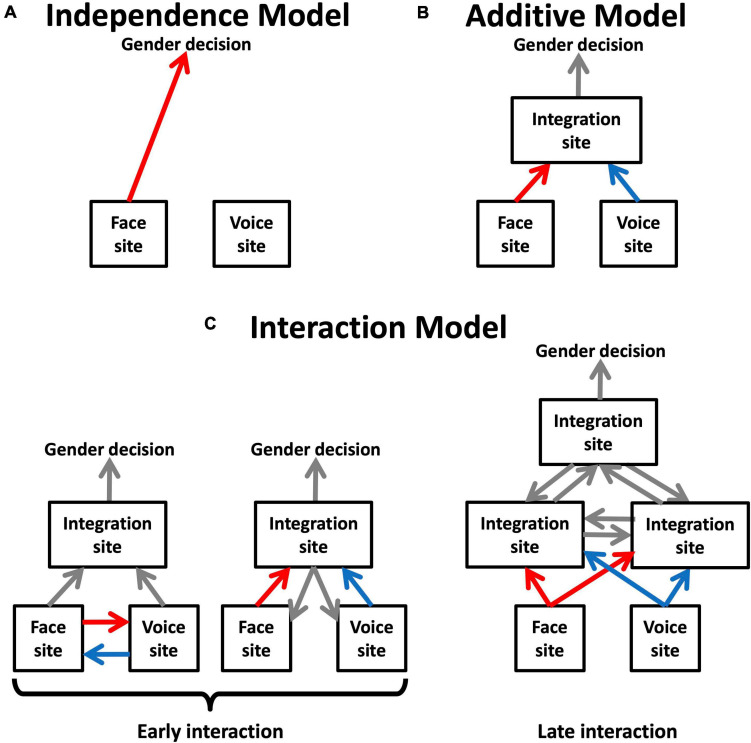
Functional models suggested by MLCM models for face-voice gender integration. **(A)** The independence model supports a direct link between unimodal face and voice sites and the site of gender decision. **(B)** The additive model implies the existence of at least one mandatory site of multimodal integration prior to gender decision. **(C)** The saturated model is compatible with several interpretations that can be divided in two groups depending on whether the non-additive interaction involves unimodal areas or takes place exclusively at a higher level.

Figure 3 presents a series of schematic networks of multimodal integration based on the type of models of inter-areal connectivity that best describe the range of possible results of the MLCM experiments. The independence model would implicate direct communication between unimodal face and voice areas and the site of gender decision, whereas its rejection would imply the existence of at least one mandatory site of multimodal integration prior to the gender decision. Models including interactions are more challenging to interpretation. The substrate of interaction effects is presumably a change in connectivity in an inter-areal network. There are two possibilities: either the unimodal areas are themselves involved in this interaction, or the changes occur exclusively at designated multimodal sites, presumably at levels hierarchically above the unimodal areas. It is not possible to decide between these two hypotheses using psychophysical experiments alone, but the question can be addressed by looking at brain activity obtained from fMRI.

Dynamic Causal Modeling (DCM) analysis was performed using MATLAB (R2014a) with SPM12 (6906). Each model was defined using the two regions of interest, FFA and TVA. DCM involves defining a series of models based on the following system of partial differential equations (Friston et al., 2003):

(9) $$f\left(x\right)=\frac{\partial x}{\partial t}=\dot{x}=\text{A}\cdot x\sum_{j}\left(u_{j}\cdot B_{j}\cdot \right)\cdot x+C\cdot u+\varepsilon y=g\,\left(x\right)+e$$

where for *i*2 areas and *j*2 inputs (visual and auditory stimulation), *x* is a vector of length *i* that describes the state of all areas at a time *t*, $\dot{x}$ is the partial derivative of *x* with respect to time; *u* is a vector of length *j* that describes the state of each input at time *t* with *u_j_* the state of a particular input; *f* is a function describing the neurodynamic mapping from the state to its partial derivative, $\dot{x},$ that depends on interactions between areas and experimental modulation; *y* is a vector of dimension *i* of the recorded BOLD signals in each area at time *t* and *g* is a function describing the mapping from states, *x*, to BOLD signals *y* depending on the hemodynamic response; ϵ and *e* are random endogenous errors; *C* is a matrix of dimensions (*i*,*j*) that describes the strength of each input to each area; *A*, a matrix of dimensions (*i*,*i*), describing the connectivity in the network that is independent of experimental manipulation of inputs; and *B_j_* correspond to a series of *j* matrices of dimensions (*i*,*i*) that describe changes in this connectivity due to experimental manipulation. Potential changes in effective connectivity are therefore captured in the B matrices.

**FIGURE 4:**
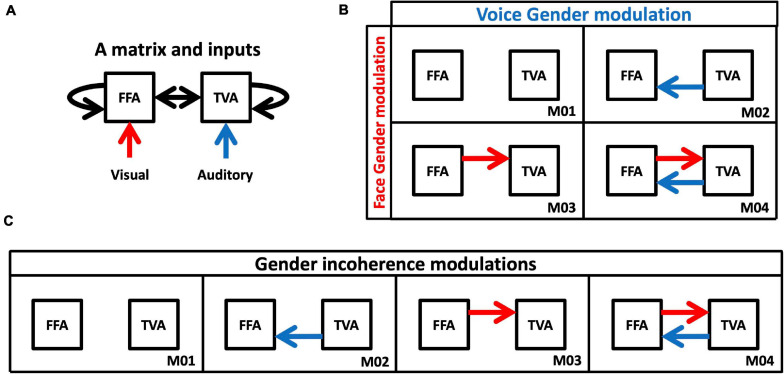
Models of DCM analysis. **(A)** Inputs and intrinsic connectivity that were applied to all cases. **(B)** Model space for changes in effective connectivity to test gender effects. Red arrows represent changes in connectivity from FFA to TVA in response to face gender (compared to gender-neutral). Blue arrows represent changes in connectivity from TVA to FFA in response to voice gender (compared to gender-neutral). **(C)** Model space for changes in effective connectivity to test congruence effects. Red and blue arrows both represent changes in effective connectivity when face and voice gender are incongruent (compared to congruent).

The first step of building DCM models involves deciding which region of interest receives which type of signal (C in Equation 9). In our case visual signals were modeled as input to the FFA and auditory signals to the TVA (red and blue arrows in Figure 4A). The second step involves defining the A matrix (A in Equation 9) describing the intrinsic connectivity in the network, which is assumed to be independent of the experimental manipulation. Given the automatic nature of face-voice integration, we considered all possible connections between and within areas (black arrows in Figure 4A). The third step is to define for each model a series of B matrices (*B*_*j*_ in Equation 9), which represent hypotheses about effective connectivity modulations between experimental conditions. Two model spaces were defined corresponding to the two tested interaction effects (Figures 4B,C):

1. Connections from FFA to TVA could either be modulated or not by adding face gender information (i.e., when faces were masculine or feminine as opposed to gender-neutral); and connections from TVA to FFA could either be modulated or not by adding voice gender information (i.e., when voices were masculine or feminine as opposed to gender-neutral). This results in the model space described in Figure 4B, with four possible models of modulation.
2. Compared to a gender congruent stimulus, connections between FFA and TVA could be modulated in either or both directions by a gender incongruent stimulus (i.e., a masculine face with a feminine voice or a feminine face with a masculine voice). This results in the model space described in Figure 4C, with four possible models of modulations.

Note that, despite their apparent similarity, the two model spaces are very different in terms of data contrasts. For example, when testing face gender modulation (i.e., models with a red arrow versus models without a red arrow in Figure 4A), activation in response to the three stimuli with a gender neutral face (and varying voice gender) is contrasted with activation in response to the six stimuli with either a masculine or a feminine face (and varying voice gender). When testing congruence gender modulation from the FFA to the TVA (i.e., models with a red arrow versus models without a red arrow in Figure 4C), activation in response to the two incongruent stimuli (with either a masculine face and a feminine voice or a feminine face and a masculine voice) is contrasted with activation in response to the seven stimuli that are either completely congruent or gender-neutral with respect to at least one modality.

For both model spaces, the four models were applied using the principal eigenvariate of the combined activation of every voxel within the FFA and the TVA of each subject. For each model within a given condition (face, voice or stimulus), model evidence, i.e., the probability of observing the measured data given a specific model, was computed based on the free energy approximation using a random-effects (RFX) analysis to account for between-subject variability (Stephan et al., 2009).

However, the analysis does not focus on the probabilities of the models *per se* but instead on the presence or absence of effective connectivity modulation from the FFA to the TVA (or TVA to FFA), while modulation in the reverse direction is controlled. Hence, we performed family comparisons, following the procedure introduced by Penny et al. (2010). First we compared a family composed of the two models in which there is no modulation from FFA to TVA to a family composed of the two models in which there is modulation (models 1 and 2 versus models 3 and 4 from Figure 4). Second we compared a family composed of the two models in which there is no modulation from TVA to FFA to a family composed of the two models in which there is modulation (models 1 and 3 versus models 2 and 4 from Figure 4). These partitions were used during the Bayesian Model Selection procedure to rank families using exceedance probability, i.e., the probability that a family is more likely than the others in a partition, given the group data.

## Results

### Psychophysics

Based on a signal detection model, perceptual scales (parameterized as *d*′) were estimated by maximum likelihood for the contributions of the two modalities to the observers’ choices in each task under the independence model described above (Equation 3) and alternative models (Equations 4, 5) in which both the voice and face signals contribute.

Each observer’s face and voice contributions were estimated initially with the additive MLCM model (Equation 4) and are displayed in Supplementary Figures 10–12. The only differences observed for male and female subjects was the voice task (linear mixed-effects model, face task: χ^2^(37) = 30.7, *p* = 0.76; voice task: χ^2^(37) = 77.3, *p* = 0.0001; Stimulus task: χ^2^(37) = 49.2, *p* = 0.09). The graph of estimated components of this small but significant difference suggests that the effect is generated by female sensitivity to strongly male faces (Supplementary Figure 13). No differences were observed between groups that judged “more masculine” or “more feminine,” after the response reversal was taken into account (linear mixed-effects model, face task: χ^2^(37) = 26.3, *p* = 0.5; voice task: : χ^2^(37) = 36.8, *p* = 0.48; Stimulus task: χ^2^(37) = 33.5, *p* = 0.63). Given these results, we combined the data sets from male and female observers who responded “more masculine” or “more feminine” for each of the three tasks in all subsequent analyses.

**FIGURE 5:**
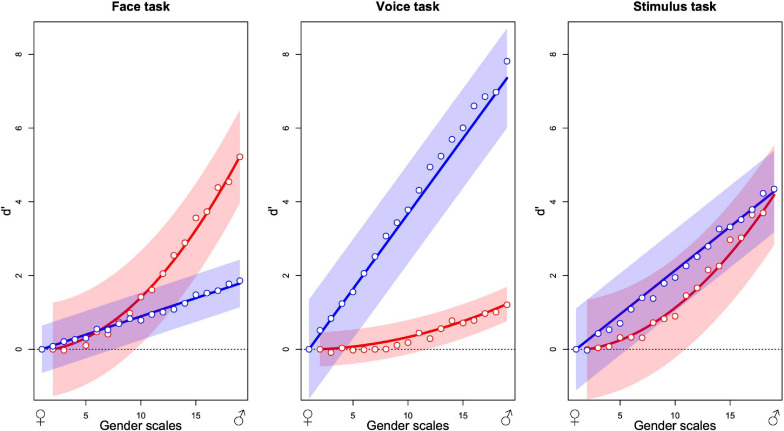
Contribution of the masculinity of the face (red) and the voice (blue) to gender decision while evaluating the gender of the face **(left)**, the voice **(center),** and the stimulus **(right)**. Abscissa indicates the levels of morphing of the faces and the voices from feminine to masculine and ordinates the contribution to gender judgment expressed as *d*′. Each plot represents the fixed effects and their 95% confidence interval from the additive GLMM analysis (lines and envelopes, respectively) and the mean values of observers corresponding to each level in the additive GLM analysis (points) for 12 observers in each panel (36 in total).

The graphs in Figure 5 summarize the additive model for each of the three tasks. Each point is the average from 12 observers. The two sets of points in each graph indicate the estimated contributions that best predict the observers’ choices over all stimulus presentations of the face (red) and the voice (blue) to the decision variable as a function of the morphing level of the stimulus, varying from most feminine (left) to most masculine (right). As an example, in the face task, the predicted internal response for a stimulus with face gender level 10 and voice gender level 15 is obtained by reading-off the ordinate value of the red point at 10 on the abscissa (about 1.5) and the blue point at 15 (about 1) and summing the two values (2.5). If the same calculation is performed for a second stimulus, for example, red point at gender 15 (about 3) and blue at 10 (about 1, summing with the other to 4), the two internal response estimates can be compared with the greater value (4) determining which stimulus is predicted to be judged as more masculine in the absence of judgment noise. The estimated scale values have been parameterized so that the standard deviation of judgment noise corresponds to 1 unit of the ordinate values. For this reason, we specify the scale as the signal detection parameter *d*′ (Green and Swets, 1966; Knoblauch and Maloney, 2012).

The contribution of each modality is task dependent. When observers judge the gender of the face, the face contribution is strongest with a smaller but significant contribution of the voice (Figure 5). When observers judge the gender of the voice, contributions invert, and when the task is to judge the gender of the stimulus, both modalities contribute about equally. In brief, effects of the task in this experiment can be conceived as relative changes of the weights of modality contributions to favor the contribution of the relevant modality.

Globally, the figures suggest that the task modulates uniquely the relative contribution of each modality without changing its functional form. For all three tasks, the voice contribution varies approximately linearly with gender level with its slope varying across tasks. Similarly, the face contribution varies in a nonlinear fashion with gender level but the form remains constant across tasks. The shape-invariance of the modality contributions (Supplementary Figure 14) shows the average values for the visual (left) and auditory (right) components for each of the three tasks, normalized to a common ordinate scale, and normalized shapes approximated with a non-linear least square approach. The voice contribution is well defined by linear and the face contribution by quadratic functions. We refitted the MLCM models to the observers’ choices with these fixed curves that varied only with respect to a task specific coefficient using a GLMM (Bates et al., 2015). The fitted curves with 95% confidence bands (Figure 5) provide a good description of the average data.

The random effect prediction for each individual (Supplementary Figures 10–12) confirms that maximum variation across subjects in face and voice tasks is found for the relevant modality while the contribution of the irrelevant modality remains lower. During the stimulus task the relative contribution across subjects of both modalities varies (Supplementary Figure 12). In fact, the 12 observers from the stimulus task fall into three separate groups: those that respond more like the observers in the face task, those that respond more like the observers in the voice task and those that tend to assign nearly the same weight to both modalities (Supplementary Figure 12). This might be due to differing strategies in performing the task, or specific inherent sensitivities to the cues from each modality that differentially influence gender judgments.

In Figure 5, the contribution of each modality, as indicated by the points in each panel, follows a similar relation as a function of gender across tasks. Only the amplitude of the response appears to vary with the change in task. This was verified by the least squares fits of the curves, which only vary in amplitude across the three panels (Supplementary Figure 14). This similarity across tasks is not imposed by the MLCM procedure for estimating the points and is not systematically observed (Ho et al., 2008). To the extent that the additive model accurately characterizes the subjects’ choices, this indicates that the top–down influence of the task does not affect overall curve shapes. It is consistent with an origin of each modality’s contribution to the decision process prior to the influence of the other modality’s signal, and more specifically from a purely unimodal source.

In the following analyses, we assumed a fixed-shape contribution from each modality, i.e., each modality’s contribution follows a parametric curve as a function of the physical morphing level on the gender scale, linear for auditory, quadratic for visual, based on the GLMM analyses above. This considerably reduces the complexity of the models as instead of estimating values for each stimulus level and combination, we only need to estimate individual coefficients that weight the entire curve shape. In particular, for the non-parametric independence model the number of parameters was one less than the number of gender levels, i.e., 17 for face and 18 for voice, but the use of a parametric curve reduces the number to only the one coefficient that controls the amplitude of the curve. For the additive model, the 37 parameters used to estimate the points in Figure 5 are reduced to 2, one coefficient for each curve. The saturated model would require 18 × 19 – 1 = 341 parameters but the use of the parametric curves reduces this number to only 3.

The independence model, for example, for the visual input becomes:

(10) $$\Delta =\beta^{V}\,\left({\left\{\phi_{1}^{V}\right\}}^{2}-{\left\{\phi_{2}^{V}\right\}}^{2}\right)+\epsilon$$

with the single parameter β^*V*^ and the exponent of 2 is included because of the quadratic shape of the face contribution for all three tasks, and an equivalent, but linear, model for an auditory input with single parameter β^*A*^. Similarly, the additive model becomes:

(11) $$\Delta =\beta^{V}\cdot \left({\left\{\phi_{1}^{V}\right\}}^{2}-{\left\{\phi_{2}^{V}\right\}}^{2}\right)+\beta^{A}\cdot \left(\phi_{1}^{A}-\phi_{2}^{A}\right)+\epsilon$$

with only two parameters.

This framework is extended to mixed-effects model by including a random effect of observer over the coefficients. The independence (visual) model, then, becomes:

(12) $$\Delta =\beta^{V}\cdot {\left({\left\{\phi_{1}^{V}\right\}}^{2}-{\left\{\phi_{2}^{V}\right\}}^{2}\right)}_{s}+b_{s}\cdot {\left({\left\{\phi_{1}^{V}\right\}}^{2}-{\left\{\phi_{2}^{V}\right\}}^{2}\right)}_{s}+\epsilon$$

The coefficient β^*V*^ is a fixed effect estimate common to every subject while the *b_S_* are random effect predictions assumed to be normally distributed with mean 0 and variance $\sigma {}_{s}^{2}$.

Generalization to the additive model is trivial but note that we did not include a random interaction term (only the random visual and auditory terms) for the mixed interaction models because the increased complexity of the random effects structure led to singular models, suggesting data overfitting (Bates et al., 2015).

Nested models were fitted and likelihood ratio tests run to re-test differences between male and female subject and between observers who judged the stimuli to be more masculine or more feminine based on the parametric curve estimations for each dimension. The results (Table 1) confirm previous findings suggesting that there are no differences between male and female observers for the face and stimulus tasks and only marginal evidence for a male/female observer difference in voice judgments, also supported by the change in AIC (face: χ^2^(2) = 1.61, *p* = 0.57; voice: χ^2^(2) = 6.6, *p* = 0.04; stimulus: χ^2^(2) = 2.25, *p* = 0.33). Moreover, there was no evidence for a difference in the fits due to the type of judgment made on any of the tasks (face: χ^2^(2) = 1.13, *p* = 0.57; voice: χ^2^(2) = 2.98, *p* = 0.23; stimulus: χ^2^(2) = 2.06, *p* = 0.36).

**TABLE 1:** Model comparison for psychophysical data.

Chisq	df	*p*	dAIC
**Masculine/feminine**			
1.1312	2	0.568	−3
2.98	2	0.23	−1
2.06	2	0.36	−2
**Male/female**			
1.61	2	0.45	−2
6.60	2	0.04	3
2.25	2	0.33	−2
**Independence/additive**			
1,425.3	2	<2.2e-16	1,421
11,335	2	<2.2e-16	11,331
5,566.5	2	<2.2e-16	5,563
**Additive/gender interaction**			
10.98	1	1e-4	9
1.20	1	0.27	−1
6.42	1	0.01	4
**Additive/congruence interaction**			
71.76	1	<2.2e-16	70
21.73	1	3 e-6	20
3.55	1	0.06	1

Likewise the independence for the additive and two interaction models described below were fitted using the parametric curves. This allowed rejection of the independence model in favor of the additive model for all three tasks (face: χ^2^(2) = 1,425, *p* < 2e-16; voice: χ^2^(2) = 11,335, *p* < 2e-16; stimulus: χ^2^(2) = 5,566, *p* < 2e-16).

The two interaction models were fitted to the data and evaluated: one in which the interaction depended on gender congruence (Equation 6) and one on gender magnitude (Equation 7). The gender magnitude interaction was tested against the additive model and found to be significant for face and stimulus tasks (face: χ^2^(1) = 10.98, *p* < 0.001; voice: χ^2^(1) = 1.2, *p* = 0.27; stimulus: χ^2^(1) = 6.42, *p* = 0.01). In contrast, an interaction due to the gender congruence of the stimuli was only significant for face and voice tasks (face: χ^2^(1) = 71.8, *p* < 2e-16; voice: χ^2^(1) = 21.7, *p* = 3.2e-6; stimulus: χ^2^(1) = 3.55, *p* = 0.06).

To summarize, instructions to judge whether the face, voice or stimulus was “more masculine” vs. “more feminine” has no influence. In addition, male and female observers performed similarly, despite a slightly larger contribution in women of the face to the voice task. After combining conditions, the independence model was rejected for all three tasks (face, voice, and stimulus comparison), indicating observers fail to completely suppress non-attended modalities. In addition, the invariance of the curve shapes suggests that unimodal sensory signals were acting *prior* to the decision process.

The two interactions tested, gender magnitude and intra-stimuli gender congruence, fit some conditions better than a simple additive model. Interestingly, gender magnitude and congruence were independent, with magnitude being non-significant for the voice task and congruent for the stimulus task. This could indicate distinct neural bases, which we explore further below with functional imaging experiments.

We examined the trial-by-trial accuracy of model predictions. Inclusion of both interactions increased the model accuracy across all three tasks (face: 78.96–79.22%, +0.26%; voice: 83.26–83.44%, +0.18%; stimulus: 80.49–80.58%, +0.09%). In other words the initial accuracies with additive models were high and the improvements in fit, while significant under the likelihood ratio tests and as indicated by the differences in AIC (Table 1) were modest. Nevertheless, improvements mirror the statistical results in that accuracy was least improved for the stimulus task.

Comparison of empirical results with simulated data in which interaction effects are modeled using the signal detection hypotheses developed in the section “Materials and Methods” and Supplementary Section 2 explores how additive and interaction model predictions differ (Supplementary Figure 9B). While the difference between additive and interaction models is much smaller than the main effects (compare ordinate scales between Figure 5 and the empirical plots of Supplementary Figure 9B), the shape of each interaction displays a distinct signature. Interactions were mostly driven by the gender magnitude effect in the stimulus task (resulting in a fan-like shape), by the congruence effect in the voice task (resulting in an inverted U shape) and by a mixture of the two in the face task. The fact that qualitatively similar changes occurred in empirical and simulated results suggests that the signal detection model needs to be modified to introduce magnitude and congruence interaction effects.

In summary, the psychophysical results show that the contributions of each modality varied according to the task by increasing the relevant and attenuating the irrelevant modality. The rejection of the independent model for all tasks means that both face and voice contribute significantly to gender evaluation in all three tasks, i.e., there are irrepressible cross-contributions of the voice during face gender evaluation and of the face during voice gender evaluation. Interestingly, the first two graphs of Figure 5 show an asymmetry; the voice contribution is higher in the voice task than the face contribution in the face task and the face contribution is lower in the voice task than the voice contribution in the face task. Finally, we found two independent interaction effects; an effect of gender congruence that was significant in the face and the voice tasks, and a multiplicative effect of gender magnitude that was significant in the face and the stimulus tasks. These effects were qualitatively compared to simulated results derived by extending the optimal cue combination model under the principles of Signal Detection theory as specified in Equations 6, 7 (see also Supplementary Section 2).

### Functional Magnetic Resonance Imaging Results

#### Regions of Interest (ROI)

**FIGURE 6:**
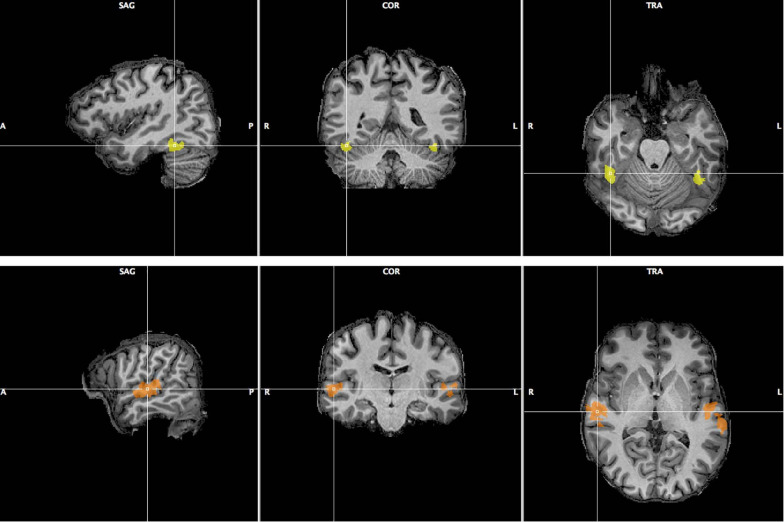
Fusiform Face Area (**top row**, yellow) and Temporal Voice Area (**bottom row**, orange) as localized in one of the 12 participants of Experiment 2. Sagittal, coronal and transverse views are shown centered around the right FFA/TVA.

We selected two areas, the FFA and TVA, activated significantly by our face and voice functional localizers, based on a GLM analysis described in the “Materials and Methods” section. Figure 6 illustrates the localization of these areas in one participant; Supplementary Figures 15, 16 show the localizations for individual participants. We computed functional signal-to-noise ratios across these ROIs for responses to the face-voice stimuli used in the main protocol. We found that the mean activity in the TVA was higher than baseline in every condition while the activity in the FFA was higher than baseline during the face and stimulus tasks but not during the voice task (Supplementary Figure 17). Scanner noise was present both during stimulation and baseline, and was therefore independent from this result. We attribute the large error bars in Supplementary Figure 17 to the small sample size used. We then explored the effects of attentional modulation of response in terms of effective connectivity between FFA and TVA using a DCM analysis.

#### Face-Voice Gender Task

So as to engage the attention of a specific modality during imaging sessions in a manner similar to that imposed by the response instructions during the psychophysical experiments, subjects performed a pre-specified gender identification task with respect to either the face, voice or stimulus on randomly signaled trials that were excluded from the subsequent imaging analyses (see Figure 2 for a full illustration of the fMRI protocol). Prior to the fMRI data analyses, we checked the results of these response trials to evaluate if participants had correctly performed the tasks.

Table 2 shows, for each task performed during the imaging experiments, the mean and standard deviation over eight subjects of the performance in stimulus identification. There were 40 response trials in total but only 30 were analyzed for each task. In particular, trials that could not be analyzed in terms of an unambiguous correct or incorrect response were excluded from analysis:

**TABLE 2:** Results of the behavioral task during fMRI recording.

	Correct	Incorrect	Miss
**Face task**			
Mean	28.50 (95%)	0.75 (2.5%)	0.75 (2.5%)
SD	0.93 (3.1%)	0.89 (3%)	0.89 (3%)
**Voice task**			
Mean	28.50 (95%)	0.50 (1.7%)	1.00 (3.3%)
SD	1.60 (5.3%)	0.53 (1.8%)	1.77 (5.9%)
**Stimulus task**			
Mean	26.88 (89.6%)	2.5 (8.3%)	0.62 (2.1%)
SD	1.25 (4.2%)	1.20 (4%)	0.74 (2.5%)

• for the face task the 10 trials with a gender neutral face;
• for the voice task the 10 trials with a gender neutral voice; and
• for the stimulus task the 10 trials with incongruent face and voice.

The column labeled “Correct” indicates the number of responses that were congruent with the gender of the attended modality (for example in the face task if the masculine sign was on the left, the face was masculine and the observer pressed the left button). “Incorrect” indicates the number of responses that were incongruent with the gender of the attended modality (for example in the previous case if the observer pressed the right button). “Miss” indicates the number of times that the observer did not respond within the 2 s limit. Participants responded in accordance with the attended modality (95%) with less than one incorrect or missed trial per acquisition. Standard deviations were low, indicating little subject variation. In summary, the evidence supports that subjects performed the task correctly, which we considered as a validation for the subsequent fMRI data analyses.

#### Dynamic Causal Modeling Analysis

Dynamic Causal Modeling with Bayesian Model Selection (Penny et al., 2010; Penny, 2012) permits evaluation of the capacity of activity in one brain area to cause or generate activity in another (effective connectivity). If such changes of effective connectivity are observed under the same conditions that we observe behavioral interaction effects, it is reasonable to conclude (1) that these changes indeed reflect a neural correlate of interaction and (2) that unimodal areas are involved.

**FIGURE 7:**
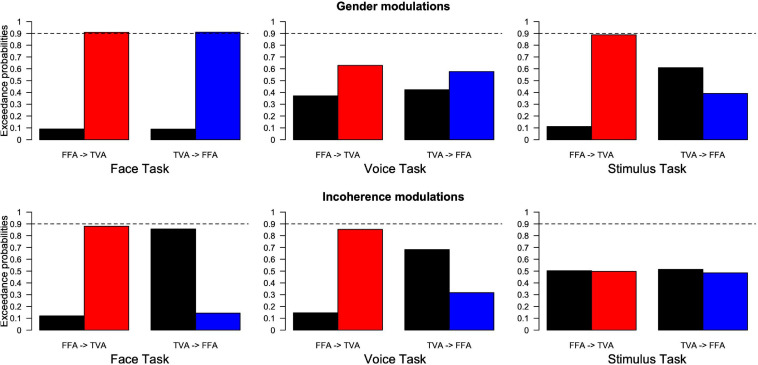
Results of family Bayesian Model Selection for all conditions, model spaces and partitions. **(Top row)** Modulations of effective connectivity by face/voice gender information. **(Bottom row)** Modulations of effective connectivity by face/voice gender incongruence. **(Left column)** Modulations when subjects attend to face gender; **(Middle column)** when they attend to voice gender; **(Right column)** when they attend to stimulus gender. Within each graph, left are the respective exceedance probabilities for the family of models without modulation from FFA to TVA (black) versus the family of models with this modulation (red). Right are the exceedance probabilities for the family of models without modulation from TVA to FFA (black) versus the family of models with this modulation (blue). Probability of 0.9 is indicated as a reference for what can be considered strong evidence in favor of a family.

Family comparison results (see section “Materials and Methods”) are shown in Figure 7, where black and colored bars indicate the exceedance probability of model families, respectively, without and with the arrow corresponding to the model spaces of Figure 4. For example, the first plot in the top left of Figure 7 corresponds to the condition where participants were instructed to pay attention to face gender in our paradigm. Its left side labeled “FFA- > TVA” illustrates the comparison (in terms of how well they describe our data) between the “family” (i.e., ensemble) of models that includes the assumption of a change in effective connectivity from FFA to TVA when face gender changes (M03 and M04 in Figure 4B, red bar) and the family of models that do not include this assumption (M01 and M02 in Figure 4B, black bar). Exceedance probability in this case represents the distribution of probabilities (e.g., 90 vs. 10%) for presence (red bar) vs. absence (black bar) of the “FFA- > TVA” assumption, given the data. Following previous work (Penny et al., 2010), we used a probability of 0.9 as strong evidence in favor of a family compared to the other but note that thresholds are less critical in this context than in a frequentist framework.

The pattern of results is similar to that obtained from the analyses of the psychophysical data. When subjects’ task was to focus on the gender of the face, the interaction effect of gender magnitude in the psychophysics experiments was mirrored by strong evidence of effective connectivity from FFA to TVA in response to face gender information (as opposed to gender-neutral faces). A similar pattern was shown in the modulation of effective connectivity from TVA to FFA in response to voice gender information (as opposed to gender-neutral voices). Similarly, there was an interaction of gender congruence in the psychophysical results and also evidence for effective connectivity modulation of FFA to TVA in response to incongruence of gender between the face and the voice. Interestingly, in addition there was evidence in favor of an *absence* of modulation (as opposed to simply no evidence in favor) from TVA to FFA in response to gender incongruence.

When the subjects’ task was to attend to the gender of the voice, there was no gender magnitude interaction in the psychophysical analyses and similarly no evidence for effective connectivity modulation in response to gender. While there was weak evidence for interaction of gender congruence in the psychophysical analysis during the face task, likewise there was weak evidence for effective connectivity modulation from FFA to TVA in response to gender incongruence.

When subjects were focusing on the gender of the stimulus, there was evidence of the gender magnitude interaction observed in the psychophysical analyses, and similarly evidence for modulation of the effective connectivity from FFA to TVA in response to face gender information. There was no interaction of gender congruence observed in the psychophysical experiments and similarly no evidence of modulation of effective connectivity in response to gender incongruence.

In summary, the results show that changes in effective connectivity parallel the MLCM interaction effects. In addition, both the gender magnitude and gender congruence interactions should be considered as early cross-modal effects as described in Figure 3 because they correspond to modulation of effective connectivity between unimodal areas.

## Discussion

Perceptual integration of face and voice information is distributed over a network of uni- and multimodal cortical (and subcortical) areas (Belin, 2017; Grill-Spector et al., 2017; Aglieri et al., 2018). While functional models of these processes have stressed the role of direct links between unimodal areas, there is extensive evidence from functional imagery for sites of multimodal integration (Young et al., 2020), raising important issues concerning the links between functional and neural models of face and voice processing. One proposition is that stable features, such as identity can be efficiently processed via unimodal sites while more transient features, such as emotional state, require higher order integration (Young et al., 2020). Here, we studied gender perception, which can be considered a stable feature and might usefully be viewed as a component of identity processing. Consistent with the above hypothesis, we found both behavioral and functional imaging evidence for cross-modal effects involving unimodal areas. As described below, however, we do not exclude a role for multimodal integration in the perceptual decision process proper.

We find that the interaction of face and voice information in gender perception can be described by a weighted combination of contributions from each modality. Further, these weights are modified by top–down influences, that is by prior instruction to subjects to base their judgments on a particular modality or on both. For example, instructing subjects to judge gender on the basis of the face led to an average fivefold ratio of the face to the voice contributions while instructing subjects to judge gender on the basis of the voice yielded a nearly eightfold ratio of the voice to face contributions. Thus, for the same stimulus set, there is a 40-fold variation in weighting due to the difference in attention, confirming that the weights are strongly influenced by top–down processes.

The MLCM technique enabled us to quantify the specific contributions of each modality. This quantification revealed that functional dependence on the relative gender of the stimulus of each modality’s response was invariant. In other words, the shape of the curve describing each modality’s contribution did not change with the top–down instruction, suggesting that the contribution of each modality depended on early unimodal pathways, and that the top–down effects could be described as a simple re-weighting of an invariant function of each modality. This result was not unexpected given that multimodal effects have been reported as early as primary visual cortex (Petro et al., 2017). Nevertheless, gender decisions could be attributed to a multi-modal combination site because both modalities contributed significantly to the decisions independently of the task (Figures 3A,B). Such a re-weighting effect (as opposed to simply increasing the contribution of the relevant modality without affecting the other) supports an optimal exploitation of limited resources.

Comparing the top–down influences of the tasks, there appears to be an auditory dominance. The auditory contribution increases more in the voice task than the visual contribution in the face task, and the visual contribution decreases more in the voice task than does the auditory contribution in the face task. Watson et al. (2013) observed the same asymmetry in a gender identification task using a nearly identical stimulus set in which they analyzed the probability of choosing one gender [but see Latinus et al. (2010)]. One explanation for the auditory dominance is that there is a greater sexual dimorphism in the auditory than the visual domain for faces; female and male voices differ significantly in fundamental frequency (Puts et al., 2011), and in the stimulus set that we used the voices obviously change in pitch when varying from female to male. Visual changes with gender tend to be widely distributed across the face and are perhaps more difficult to define (Macke and Wichmann, 2010). With the current stimulus set, our subjective impression was that gender differences appear to be related to the sharpness of contours, which is in accordance with previous results linking face gender perception and contrast (Russell, 2009). However, if the differences in the sensory ranges between modalities were so large, we might expect the auditory signals to dominate in the stimulus task, which is not the case; instead both dimensions contribute about equally. Additionally, the response range along the *d*′ scale of the visual component in the face task is only about one-third smaller than that for the auditory component in the voice task. These observations suggest that integration mechanisms are capable of adjusting modality specific weights to compensate for differences in the range of input signals as has been demonstrated in anomalous color vision (MacLeod, 2003; Knoblauch et al., 2020; Tregillus et al., 2020).

The additive model provided a good overall account of the data, predicting observers’ choices with on average 80% accuracy across all conditions. However, small but significant improvements were obtained by including specific interaction effects related to the congruence of gender between modalities and to the magnitude of the gender signal (i.e., differences from neutral gender) within each modality. We simulated these interaction effects in terms of contributions to decision noise based on an optimal cue combination decision rule within a signal detection model and found that simulated results agree qualitatively with the estimates from the data (Supplementary Section 2). According to these simulations, when the gender information across modalities is inconsistent, the integration process has an increased variance leading to a decision bias. For the gender magnitude interaction, the closer the gender within a given modality is to neutral, the larger the variance assigned to it, which similarly leads to decisional biases.

The two interaction effects do not manifest under the same conditions. Significance of the congruence interaction occurs during the face and the voice tasks; significance of the gender magnitude interaction occurs during the face and stimulus tasks. One interpretation of the lack of significance of the congruence interaction during the stimulus task is that there is a stronger prior applied in this task to assume that the gender cues from face and voice are congruent, and thus, to base the judgments only on the within modality differences, while ignoring the cross modality conflicts. An alternate possibility is that congruence depends on the task relevance of the modality, so that congruence is ignored when both modalities are relevant. In the case of the gender magnitude interaction, supposing that face gender is more ambiguous than voice gender, for the reasons stated above, observers may be more likely to be influenced by the gender magnitude when the face is a relevant factor, i.e., in the face and stimulus task, compared to when it is not as in the voice task.

These results led us to explore the effective connectivity between cortical areas implicated in face and voice processing for neural correlates of the interactions. Using an equivalent face-voice gender categorization task with DCM, we found that modulations of connectivity between the FFA and the TVA mirrored the behavioral interaction effects, consistent with the hypothesis that these effects do not exclusively depend on higher-order multi-modal integration sites (Figure 3C).

Mirroring the conditions in which the congruence interaction occurs, the effective connectivity from FFA to TVA was modulated for the face and voice tasks. Such an effect is not observed during the stimulus task, perhaps reflecting the reduced weight assigned to the auditory component observed in the psychophysical experiments and its minimized role in integration in the presence of gender incongruence across modalities.

There was no impact on effective connectivity with changes in gender magnitude from neutral during the voice task but there was a modulation from FFA to TVA and from TVA to FFA during the face task as well as a modulation from FFA to TVA during the stimulus task. These findings parallel the psychophysical results for the gender magnitude interaction, which, also, was significant for the face and stimulus tasks but not for the voice task. In the case of the stimulus task, this might represent a mechanism generating a change in weight in both modalities to equalize their contributions to the judgments. Given the default auditory dominance described above in the psychophysics, a similar explanation might apply in the face task. We hypothesize that task-dependent reweighting of the contributions of the modalities constitutes a behavioral correlate of the task-dependent dynamic switching of inter-areal hierarchical relations observed in macaques (Bastos et al., 2015).

The two interactions modeled in the psychophysical analyses correspond to two types of discrepancies in the cues that must be processed in order to make a gender judgment. In the case of the congruence interaction, there is a conflict between voice and face gender identity. On the other hand, the gender magnitude interaction depends on the strength of the gender signal and, thus, could be related to the precision of its encoding (or lack thereof).

These differences could be related to the multiplicity of feedback pathways (Markov et al., 2014) having distinct functional roles (Markov and Kennedy, 2013; Roelfsema and de Lange, 2016; Shipp, 2016; Bergmann et al., 2019).

One computational theory about the role of feedback signals is that they contribute to the construction of generative models of the outside world (Mumford, 1992; Rao and Ballard, 1999; Friston, 2002, 2005). In this framework each processing step is conceived as striving to predict its feedforward inputs based on the feedback signals it receives. Predictions (which can be interpreted as an internal representation) are transmitted to lower levels via feedback signals, and prediction errors (which can be interpreted as a function of model residuals) are transmitted to upper levels via feedforward signals. In this context a conflict in the integration of two signals would lead to an error signal propagated from lower to higher areas. In this framework, gender incongruence modulation in which there is an increase in effective connectivity from FFA to TVA when face and voice genders are different can be interpreted as an error signal.

Anatomically, the FFA and TVA belong to streams of two different modalities, making their hierarchical relations indirect. Support for the hypothesis that the TVA is hierarchically higher than the FFA can be found in the macaque where a face responsive patch comparable to the FFA is situated in the TEpd area (Lafer-Sousa and Conway, 2013) and voice responsive neurons that have been argued to form a TVA-like patch (Perrodin et al., 2011; Belin et al., 2018) are situated in the parabelt area. Analysis of anatomically derived measures of hierarchy that are based on laminar connectivity patterns (Markov et al., 2014) indicate that the TEpd is indeed hierarchically lower than the parabelt area PBr.

Current studies indicate that feedforward and feedback influences are mediated through distinct signaling channels across the cortex (Bastos et al., 2015, 2018) and via laminar connectivity, through distinct anatomical pathways (Markov et al., 2014). A natural progression of the current work would be to investigate the layer-specific modulations of the interactions revealed here using high resolution, laminar resolution fMRI (Lawrence et al., 2019), a method that has recently been used to study visuo-auditory processes (Gau et al., 2020).

The present findings are paradoxical as they suggest that unimodal top–down influences modulate the weight of visual and auditory contributions in a simple gender perception task rather than leading to a perceptual blending or integration of auditory and visual responses, an issue that was previously suggested by Clark (2013). Many previous studies have examined feedback on early sensory areas, such as V1, with respect to low level stimulus features encoded early in the hierarchy, such as orientation (De Lange et al., 2018). For example, error signals can be generated by manipulating the subject’s expectation of these features across trials. Here, we studied a multi-modal gender perception task in which error signals were generated by providing conflicting information to one modality while subjects made decisions based on information from a different modality, thus allowing us to probe such interactions among higher order areas. The results are relevant to current hypotheses on the generative role of feedback pathways, which are speculated to relay expectations and ensure inference of the causes of sensory stimulus (Rao and Ballard, 1999; Bastos et al., 2015) and emphasize the generative role of multiple feedback processes in cross-modal interactive processes that are highly distributed at low levels of the cortical hierarchy, coherent with the high-density of the cortical graph (Vezoli et al., 2021).

## Data Availability Statement

The data sets generated and analyzed in the current study are available on request from author CA. fMRI data are located on the repository https://shanoir.irisa.fr.

## Ethics Statement

The studies involving human participants were reviewed and approved by Comité de Protection des Personnes Sud-Est III Groupement Hospitalier Est, Hôpital Civil de Lyon Bron, France. The patients/participants provided their written informed consent to participate in this study.

## Author Contributions

HK and KK: project conception and procured funding. CA, PG, and KK: the experimental design. CA and PG: data collection. CA, PG, KB, and KK: data analysis. CA, PG, HK, and KK: wrote the manuscript. All authors revised successive versions of the manuscript.

## Conflict of Interest

The authors declare that the research was conducted in the absence of any commercial or financial relationships that could be construed as a potential conflict of interest.
